# Histological and Metabolic State of Dams Suckling Small Litter or MSG-Treated Pups

**DOI:** 10.1155/2016/1678541

**Published:** 2016-11-27

**Authors:** Claudia Regina Capriglioni Cancian, Nayara Carvalho Leite, Elisangela Gueiber Montes, Stefani Valeria Fisher, Leticia Waselcoski, Emily Caroline Lopes Stal, Renata Zanardini Christoforo, Sabrina Grassiolli

**Affiliations:** ^1^Department of General Biology, University of Ponta Grossa, Ponta Grossa, PR, Brazil; ^2^Department of Structural and Functional Biology, University of Campinas, Campinas, SP, Brazil; ^3^Department of Physiology, University of West Parana, Cascavel, PR, Brazil

## Abstract

Lactation is an important function that is dependent on changes in the maternal homeostasis and sustained by histological maternal adjustments. We evaluated how offspring manipulations during the lactational phase can modulate maternal morphologic aspects in the mammary gland, adipose tissue, and pancreatic islets of lactating dams. Two different models of litter-manipulation-during-lactation were used: litter sizes, small litters (SL) or normal litters (NL) and subcutaneous injections in the puppies of monosodium glutamate (MSG), or saline (CON). SL Dams and MSG Dams presented an increase in WAT content and higher plasma levels of glucose, triglycerides, and insulin, in relation to NL Dams and CON Dams, respectively. The MG of SL Dams and MSG Dams presented a high adipocyte content and reduced alveoli development and the milk of the SL Dams presented a higher calorie and triglyceride content, compared to that of the NL Dams. SL Dams presented a reduction in islet size and greater lipid droplet accumulation in BAT, in relation to NL Dams. SL Dams and MSG Dams present similar responses to offspring manipulation during lactation, resulting in changes in metabolic parameters. These alterations were associated with higher fat accumulation in BAT and changes in milk composition only in SL Dams.

## 1. Introduction

Lactation is a critical period in female life that requires a coordinate adaptation in energy metabolism. This period induces changes in morphologic aspects, particularly in adipose and glandular tissues to sustain milk production [1, 2]. As such, the health and nutritional status of the mother have an influence on milk composition, which in turn affects neonate health. For example, obesogenic maternal diets, with an excess of nutrients, such as lipids or carbohydrates, can lead to changes in milk volume [3, 4] and milk composition, affecting lactose [5] and fat content [5, 6]. In this regard, mice that are chronically exposed to a high fat diet present a reduction in mammary gland (MG) area, high leptin levels, and systemic prolactin resistance, which lead to impaired lactation performance [7]. Moreover, maternal obesity or undernutrition, during lactation, affects offspring growth, predisposing them to the development of obesity and cardiovascular disease in adult life [8, 9].

An increase in lipolysis in the adipose tissue occurs during lactation, mobilizing fat stores accumulated during gestation as energetic substrates for milk synthesis. Studies in rodents have confirmed that changes in glucose and lipid metabolism occur during lactation and that these are accompanied by morphological adaptations in white adipose tissue (WAT), brown adipose tissue (BAT), and MG [10]. Moreover, basal glucose utilization is reduced in WAT and muscle during lactation, indicating changes in insulin sensibility or its action; events associated with diminished plasma levels of insulin and glucose are frequently observed in this phase [11].

In rodents, lactation is also characterized by changes in BAT function and morphology. During this period, decreased thermogenesis occurs in the BAT and this is frequently associated with tissue hypotrophy, reduced mitochondrial respiration and content, and diminished sympathetic activity [12, 13]. Additionally, several studies have shown that milk production is significantly correlated with BAT function and the gene expressions of uncoupling proteins (UCP1 and UCP3), which result in downregulation of BAT thermogenic activity. These adjustments in BAT function occur as an energy-sparing mechanism to maximize energy availability for milk production [14].

All of these morphological and metabolic adjustments are necessary to sustain milk production, which result in changes in MG structure and metabolism. Diet composition, hormones, and nursing stimuli affect MG growth during lactation [15]. As such, litter size affects MG in lactating rats [16] and maternal diet during lactation alters lactation capacity and MG morphology [17]. The suckling period is a critical window of development, where nutritional and hormonal maternal states have a direct impact on offspring development and health [18]. Here, we used two different models of offspring manipulations during the suckling period and evaluated their impact on maternal conditions. In the first model, we used a well recognized model of different litter sizes to study the effects of postnatal overfeeding. Thus, rats were raised in small litters (SL, 3 pups/dam) or normal litters (NL, 9 pups/dam), representing a model of metabolic programming induced during lactation, related to a greater incidence of obesity and diabetes in adult life [19]. In the second model, the litter size was maintained similar in two groups (6 pups/dams); however, glutamate monosodium (MSG) was administered to half of the pups to induce hypothalamic lesions and promote obesity in adult life [20]. Thus, we employed different models of offspring manipulations during the lactation phase to improve our understanding of how the profile of pup suckling can induce changes in the maternal state, reflected in milk composition. It is well recognized that the synergisms between dams and pups that occur during lactation can determine the health of offspring later in life; however studies exploring the maternal conditions in these situations are scarce. Studies in humans and rodents suggest an association between the duration of lactation and a reduced maternal risk of metabolic disease, by a mechanism probably related to effect of lactation on reestablishing glucose homeostasis after delivery [21]. Thus, we herein investigated how offspring manipulation during the suckling period can induce maternal morphological changes in the adipose tissue, mammary gland, and endocrine pancreas, as well as their effects on maternal plasma metabolic parameters and milk composition.

## 2. Results

**Figure 1:**
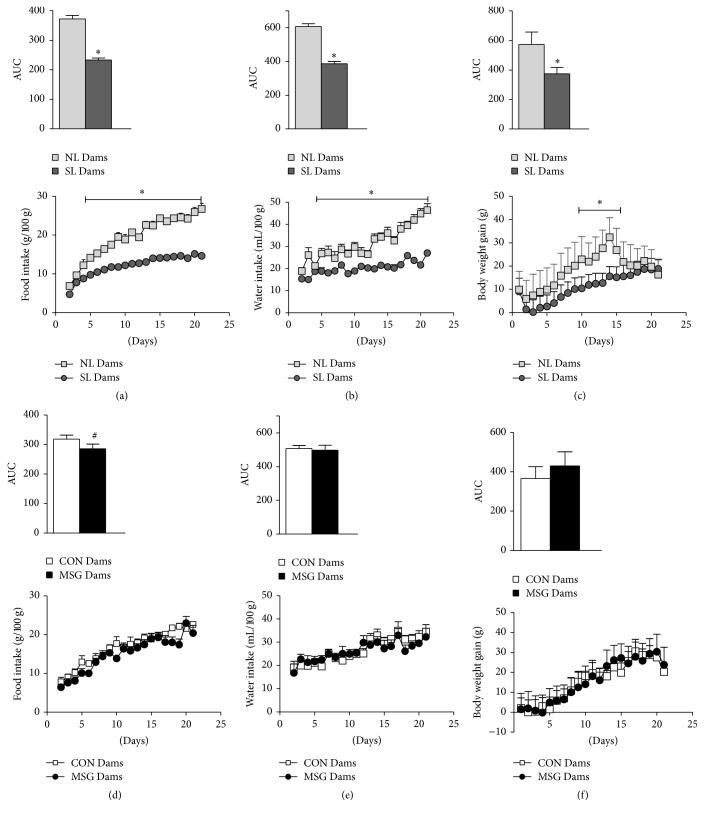
Food intake, water intake, and body weight gain in lactating dams. Food intake (a, d), water intake (b, e), and body weight (c, f) of lactating dams from the 1st to 21st day of lactation. Data are the means ± SEM (*n* = 8–10 dams/group). Symbols above the bars represent statistical differences according to Student's* t*-test (*p* < 0.05). ^**∗**^NL Dams* versus* SL Dams; ^**#**^CON Dams* versus* MSG Dams.

Results are presented comparing NL Dams* versus* SL Dams and CON Dams* versus* MSG Dams. The patterns of body weight and food and water intake changes in dams during lactation are shown in Figures 1(a)–1(f) with the respective area under curve (AUC) above. Significant reductions in food and water intake and body weight were observed in SL Dams from day 5 until day 21 of lactation, compared to NL Dams. Thus, the AUCs of food, water, and body weight gain were reduced by 37.4%, 36.2%, and 53.4%, respectively, in the SL Dams, compared to the NL Dams (*p* < 0.05). In contrast, only the AUC of food intake (Figure 1(d); showed above) was reduced in MSG Dams by 10.3% in relation to the CON Dams, *p* < 0.05.

Table 1 shows that pup manipulations alter blood biomarkers in dams at 21 days of lactation. SL Dams presented increased levels of plasma glucose (33.4%), insulin (46.7%), and triglycerides (43.7%) in relation to the NL Dams. Similarly, the MSG Dams presented higher plasma glucose (35.1%), insulin (69.3%), and triglycerides (73.1%), compared to the CON Dams (*p* < 0.05). Milk composition was evaluated at 21 days of lactation, as shown in Table 2. Milk from SL Dams presented a higher caloric value (32.2%), compared to the milk from NL Dams, probably due to the higher fat content (44.4%) in this milk, compared to milk from NL Dams (*p* < 0.05). These parameters were not significantly affected in the MSG Dams in relation to the CON Dams. Milk protein content was not altered in either the MSG Dams or the Dam SL, compared, respectively, to CON Dams and NL Dams. However, leukocyte numbers were elevated (53.7%) in the milk of MSG Dams, compared to milk from the CON Dams (*p* < 0.05).

Since litters manipulation during lactation affected milk composition, our next step was to assess the body weight of offspring at post weaning (21 days) and in adult life (90 days); to evaluate obesity, adult offspring were weighed and fat content was determined (Table 3). The body weight of SL offspring was 13.6% and 19.9% higher at 21 and 90 days, respectively, in relation to NL offspring rats. The MSG offspring presented a reduction of 10.6% in body weight, compared to CON offspring only in adult life (90 days). Visceral adipose tissue was 27.0% and 118.7% higher in adult SL offspring and adult MSG offspring rats, compared to adult NL offspring and adult CON offspring groups, respectively (*p* < 0.05).

**Figure 2:**
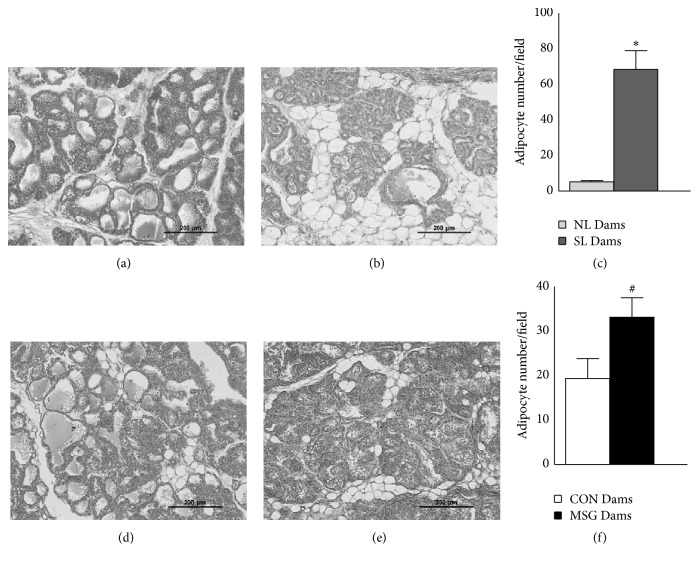
The effect of offspring manipulations on mammary gland histology in lactating dams at 21 days of lactation. Representative photomicrographs (magnification 200x; scale bar, 200 *μ*m) of H&E-stained mammary gland tissue from lactating dams at 21 days of lactation: ((a)-(b)) NL and SL, respectively, and ((d)-(e)) CON and MSG, respectively. Quantitative analyses of adipocyte numbers in mammary glands are represented in (c) and (f). The data are mean ± SEM of 20 sections collected from at least 3 dams/group. Symbols above the bars represent statistical differences in Student's* t*-test (*p* < 0.05). ^**∗**^NL Dams* versus* SL Dams; ^**#**^CON Dams* versus* MSG Dams.

The effects of offspring suckling on MG morphology at 21 days of lactation are shown in Figures 2(a)–2(e). In the quantitative histological analysis, we observed that the MG of SL Dams and MSG Dams presented a reduction in the mammary alveolus in the parenchymal tissue, where the individual alveoli show irregular aspects and a reduction in alveolar lumen, in relation to those of the NL Dams and CON Dams groups, respectively. In addition, our quantitative analysis demonstrated that SL Dams and MSG Dams also presented elevated adipose tissue content in MG (Figures 2(a)–2(f)). In this respect, the adipocyte content in the MG of SL Dams and MSG Dams was found to be increased by 1215% and 41.4%, compared to that of the NL Dams and CON Dams, respectively (Figures 2(c) and 2(f); *p* < 0.05). Interestingly, we observed that changes in the above-mentioned alveoli structure and in adipocyte content in the MG were more accentuated in MG from SL Dams.

**Figure 3:**
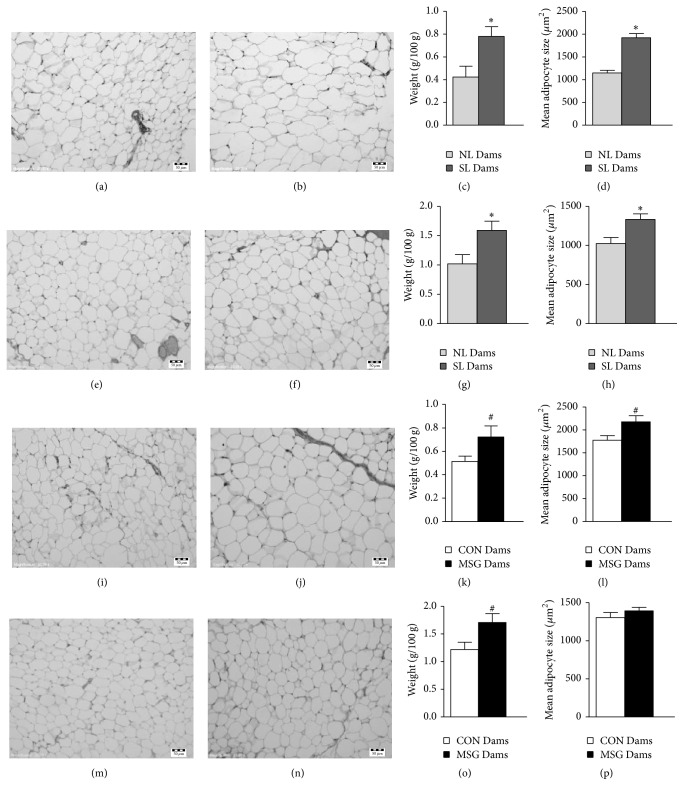
Morphologic adaptations of WAT in dams at 21 days of lactation. Representative photomicrographs (magnification 200x; scale bar, 50 *μ*m) of H&E-stained sections of retroperitoneal, (a) (NL), (b) (SL), (i) (CON), (j) (MSG) and ovarian adipose tissue, (e) (NL), (f) (SL), (m) (CON), and (n) (MSG). The quantification of mean adipocyte areas (mean ± SEM) is depicted in (d), (h), (l), and (p). Sections were evaluated from *n* = 3 dams/group and three image fields per section. Retroperitoneal fat content is represented in (c) (NL, SL) and (k) (CON, MSG) and ovarian fat content is represented in (g) (NL, SL) and (o) (CON, MSG). Symbols above the bars represent statistical differences in Student's *t*-test (*p* < 0.05). ^*∗*^NL Dams* versus* SL Dams; ^**#**^CON Dams* versus* MSG Dams.

**Figure 4:**
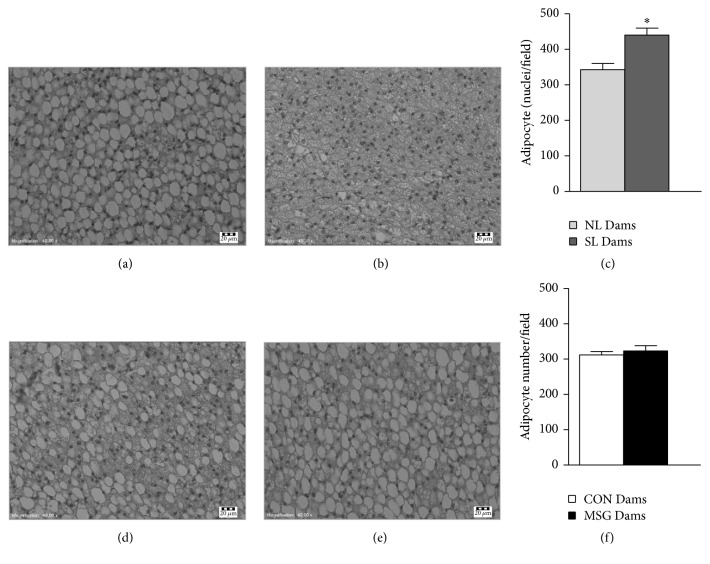
Impact of offspring manipulations on BAT morphology in lactating dams at 21 days of lactation. Representative photomicrographs (magnification 400x; scale bar, 20 *μ*m) of H&E-stained sections of interscapular BAT from lactating dams (a, b, d, and e). Quantification of adipocyte proliferation evaluated by nuclei counts (c and f). Data are median ± SEM; sections were evaluated from *n* = 3 dams/group and three image fields per section. Symbols above the bars represent statistical differences according to Student's* t*-test (*p* < 0.05). ^*∗*^NL Dams* versus* SL Dams.

The WAT content and adipocyte size in dams at 21 days of lactation are shown in Figure 3. SL Dams and MSG Dams presented increases of approximately 60.2% in retroperitoneal and ovarian fat depots, in relation to the NL Dams and CON Dams, respectively (Figures 3(a)–3(p), *p* < 0.05). The adipocyte areas in the retroperitoneal depots were 67.4% higher in SL Dams, compared to NL Dams, and 22.6% higher in MSG Dams, compared to CON Dams (Figures 3(d) and 3(l), *p* < 0.05). Similar alterations were observed in the adipocytes of the ovarian depots of the SL Dams, compared to the NL Dams (Figure 3(h)). At 21 days of lactation, BAT proliferation was higher (28%) in SL Dams, in relation to the NL Dams (Figure 4(c); *p* < 0.05).

Morphologic alterations were not observed in ovarian adipose tissue and BAT in MSG Dams, compared to those of the CON Dams (Figures 3(m)–3(p) and 4(d)–4(f)).

**Figure 5:**
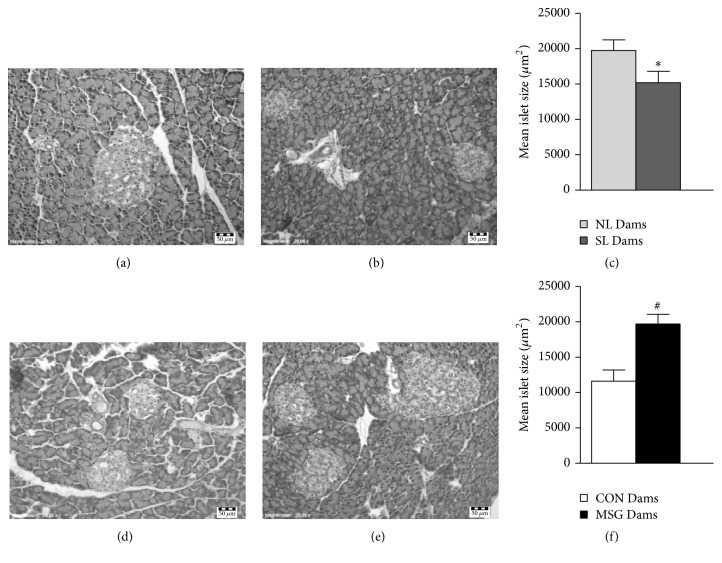
Adaptations in pancreatic islet histology in lactating dams at 21 days of lactation. Representative photomicrographs (magnification 200x; scale bar, 50 *μ*m) of H&E-stained sections of pancreatic islets from lactating dams (a, b, d, and e). (c, f) Quantification of islet size (*μ*m^2^); data are median ± SEM. Sections were evaluated from *n* = 3 dams/group and three image fields per section. Symbols above the bars represent statistical differences according to Student's* t*-test (*p* < 0.05). ^**∗**^NL Dams* versus* SL Dams; ^**#**^CON Dams* versus* MSG Dams.

Finally, we evaluated the impact of offspring manipulation on pancreatic islet histology in dams at 21 days of lactation (Figure 5). The diameters of islets from SL Dams were 23.6% smaller in relation to islets from NL Dams (Figure 5(c)). In contrast, diameters of islets from MSG Dams were increased by 69.8%, compared to islets from CON Dams (*p* < 0.05) (Figure 5(f)).

## 3. Discussion

Lactation is the most energetically demanding period of life in the female mammal, inducing adaptations in metabolism, including changes in the food intake control [1, 2]. Thus, hyperphagia is an evident characteristic of the lactation phase, an event attributed to changes in the central action of hypothalamic neuropeptides linked to the regulation of food intake [22–24]. Our study found a reduction in food intake in lactating SL Dams* versus* NL Dams, probably due to the lower energetic expense needed to sustain the reduced number of pups during suckling. These data confirm results also obtained by Zhao et al. 2013 [25], which show that litter size and pup growth have a direct impact on maternal food intake control and milk production during lactation. Interestingly, we observed that MSG Dams that present similar pup numbers in relation to CON Dams also presented a slight reduction in food intake, suggesting an alteration in the suckling of MSG-treated pups. It is well established that postnatal MSG treatment produces hypothalamic neurotoxic cell destruction, damaging the arcuate nucleus and promoting obesity in adult life [26, 27]; however, studies on pups of rats treated with MSG during the lactation phase are scarce and contradictory. A study realized by Schoelch et al. 2002 [28] showed that MSG-treated pups present an enhanced energy expenditure and simultaneous reduction in suckling milk intake, corroborating our hypothesis that MSG-treated pups undergo changes in suckling stimulus that affect the energy expenditure in lactating MSG Dams and resulting in reduced food intake. Whether hypothalamic lesions induced by MSG treatment can alter neural control during suckling is unknown.

During the lactation period, glucose and lipid homeostasis are modified, due to the changes in insulin secretion and action, promoting a reduction in lipid synthesis and basal glucose utilization in WAT and muscle [29]. These events are associated with the lower blood glucose and plasma insulin concentrations observed in this phase. Interestingly, human and rodents studies performed during the lactation period suggest an association between the duration of lactation and a reduced maternal risk of metabolic disease, mechanism probably related to the effect of lactation on reestablishing glucose homeostasis after delivery [21]. Corroborating these data, in our study, SL Dams and MSG Dams presented elevated blood levels of glucose, insulin, and triglyceride levels at 21 days of lactation, suggesting an excess of nutrients that are probably not used in milk synthesis. In addition, a deterioration in the regulation of glucose metabolism has been found in animals that had repeated pregnancies without lactation [30]. These findings indicate that the offspring manipulations carried out during our study probably reduce milk synthesis in MG, affecting their structure. As such, the histological analysis in our study confirms that SL Dams and MSG Dams present reduced MG development and, probably, milk synthesis. In this regard, studies have demonstrated that the metabolic activity of the MG is a major factor in plasma insulin regulation during the suckling period via a mechanism involving prolactin, the primary hormone in the milk synthesis, and with a known effect on insulin secretion and synthesis [31, 32]. Moreover, the sealing of all teats in dams or litter removal during lactation produces increased plasma insulin levels in lactating dams [33, 34]. Despite the similar plasma metabolic profile found in lactating SL Dams and MSG Dams, the milk composition was affected by offspring manipulation at 21 days of lactation. Milk from the SL Dams presented a high caloric percentage, probably due to the elevated fat content. These data confirm some studies that have shown that small litters of rat pups reduce the competition for milk, resulting in increased milk consumption and milk rich in lipids, especially triglycerides [19, 35]. Unfortunately, the mechanism that explains this elevated milk triglycerides content is not well characterized. The milk composition promotes a metabolic program that affects body weight control. Thus, according to our data, rodents from small litters are overweight at weaning.

On the other hand, the milk composition of lactating MSG Dams was not modified, suggesting that milk composition changes are not responsible for MSG obesity in adult life. We observed that MSG-treated pups were not obese at 21 days of life, confirming data obtained by [36]. In this obese model, evident fat accumulation, insulin resistance, and glucose intolerance are present at 60 days of life [37]. However, contradictory results from milk ingestion in MSG-treated pups were found by Schoelch et al. 2002 [28] and Miyata et al. 2009 [38]. The effects of MSG-treated pups on the milk composition in lactating dams have not been determined. Interestingly, milk from the MSG Dams also presented increased leukocyte contents. Further studies are necessary to evaluate the effects of these changes in lactation on MSG-treated pups.

As such, we have demonstrated that offspring manipulations during lactation change the morphology of the MG at 21 days of lactation, modifying parenchymal tissue, decreasing numbers of intact alveolar units, and simultaneously increasing adipocyte content in the MG, which could mean a reduction in the functional activity of MG and a reduction in milk synthesis. In addition, the greater adipose tissue content in MG observed in our study could affect lactation. This hypothesis is corroborated by review compiled by [39], showing that mammary adipose tissue produces locally effective concentrations of estrogen in obese women, which could alter MG development and suckling performance. Moreover, the suckling stimulation, particularly due to litter size, has an effect on the growth of suckled MG [15, 40], possibly explaining the reduced development of the MG observed in lactating SL Dams. Similar histology adjustments were also observed in MG from lactating MSG Dams. However, in this model, the reduced development of the MG is probably promoted by other factors, such as changes in suckling stimulus, frequency, or intensity of lactation, since the litters of MSG Dams and CON Dams were similar. Studies on MSG-treated pups and milk ingestion are contradictory [28, 38]; further studies are necessary to clarify the impact of neonatal MSG treatment on suckling performance.

During lactation in rats, the storage of lipids in adipose tissue stops and lipid uptake in mammary tissue increases to permit lipid transference to milk [2, 41, 42]. In the present study, we observed an increase in WAT content, accompanied by adipocyte hypertrophy in lactating SL Dams and MSG Dams. These findings are probably attributed to the elevated insulin levels also observed in both lactating Dams groups as this hormone has known lipogenic actions [43]. In addition, studies have found that maternal obesity during lactation determines the health of offspring, predisposing them to the development of obesity and metabolic syndrome in adulthood [8, 44]. We demonstrated that, at 90 days of life, both MSG-rat offspring and SL-rat offspring presented obesity, characterized by high WAT content.

Lactation is associated with an adaptive decrease in BAT thermogenesis, an event necessary for milk production in rodents [45, 46]. During lactation, the thermogenic activity and capacity of BAT are substantially reduced, possibly due to suckling-induced suppression of the sympathetic axis to BAT [47]. We also evaluated BAT morphological adaptations in dams at 21 days of lactation. Interscapular BAT from SL Dams presented increased adipocyte proliferation and reduced lipid droplet accumulation, indicative of greater BAT thermogenesis compared to NL Dams. It is known that thermogenic processes in BAT are suppressed in dams nursing large litters [13]. Thus, SL Dams nursing small litters probably increase BAT thermogenesis. Despite this characteristic, the SL Dams presented a high WAT accumulation, suggesting that increased BAT function is not sufficient for burning off excess energy. Lactating MSG Dams did not present any alterations in morphological aspects in the BAT.

Finally, we evaluated the impact of offspring manipulations on islet histology in lactating dams at 21 days of life. SL Dams presented a reduction in islet size, compared to NL Dams, while increased islet size was observed in the pancreas of MSG Dams compared to CON Dams. Previous reports showed an increase in pancreatic weight during lactation, as well as hypertrophy of the beta cells [48]. According to these authors, these adaptations may be attributed to elevated prolactin levels. Thus, we suggested that a reduced suckling stimulus in SL Dams occurs, in turn reducing prolactin levels and inhibiting the normal lactational hypertrophic prolactin action frequently observed in the endocrine pancreas. On the other hand, the hypertrophy observed in islets from MSG Dams appears to indicate an increase in prolactin levels. Unfortunately, we did not evaluate milk ingestion in MSG pups; however Miyata et al. 2009 suggested that MSG-treated pups ingest more milk in the long term, compared to controls [38], indicating an altered suckling performance in MSG pups. This situation could reflect in prolactin secretion and consequently affect the endocrine pancreas. Accordingly, histological analysis in MG from MSG Dams suggested reduced functional activity. Further studies are required to focus on plasma prolactin levels to confirm this hypothesis in both the models of lactating dams used in this study.

In conclusion, the results of present study confirm that the maternal conditions of lactating dams with small litters and of lactating dams suckling MSG-treated pups are different to those of the normal lactational state. In these lactating dams, changes were observed in body weight composition and corporal fat mass, in association with hypertrophic adipocytes and altered blood metabolic profile, events probably associated with a reduction in lactational activity, as suggested by the atrophic aspect of the MG found in the histological analysis. On the other hand, specific morphologic adjustments in BAT and the endocrine pancreas were observed only in lactating dams suckling a small litter, events that could be related to the changes in milk composition found in these dams and not observed in milk from MSG Dams.

## 4. Materials and Methods

### 4.1. Animals and Experimental Design

Virgin female Wistar rats (*Rattus norvegicus*) were mated at 8 weeks of age in the ratio (3 female/1 male). Pregnant rats (*n* = 20) were placed in individual cages until the birth of the litters (day zero of lactation), when the dams were divided into two experimental groups. In the first model (*n* = 10 dams), the pup number in the litter was normalized to ten on day 1 of lactation. On day 3, the litters were adjusted to 9 pups/dam for the normal litters (NL) and to 3 pups/dam for the small litters (SL). The rats growing up in small litters were used to model early postnatal overnutrition, a known model of metabolic programming [49] that promotes obesity in rats in adult life. In the second model (*n* = 10 dams), the litter number was normalized to six on day 2 of lactation. On the 2nd day, half of the rats of litter were submitted to hypothalamic lesions induced by the subcutaneous injection of monosodium glutamate (MSG, 4 g/Kg/5 consecutive days). Equimolar saline solution was administered to the control group (CON). This protocol induces damage to the arcuate hypothalamic nucleus, which promotes obesity in adult life, as demonstrated by several studies [20, 26]. In both experimental groups, pups were weaned at 21 days of lactation. The effect of litter size manipulations during lactation on the maternal state was investigated for each experimental group, comparing SL Dams* versus* NL Dams and MSG Dams* versus* CON Dams at 21 days of lactation. All dams were housed individually and maintained under a 12 h light/12 h dark and constant temperature (21 ± 2°C). Food (Nuvital®, Curitiba, Paraná, Brazil) and water were provided* ad libitum*. The study followed the Brazilian guidelines for the protection of animals used for experimental purposes and was conducted with the approval of the Ethical Committee for Animal Experiments for the State University of Ponta Grossa (CEUA, Protocol no. 03482/2012).

### 4.2. Body Weight and Food and Water Intake

Lactating dams were weighed every day from the 1st day until the 21st day of lactation. In the same period, food intake and water consumption were also measured daily. Pups were also weighed at 21 and 90 days of age.

### 4.3. Milk Analyses

Milk samples were collected from lactating dams at 21 days of lactation between 10.00 and 13.00 h to avoid diurnal variation in milk composition and 4 hours after pups had been removed. For milk collection, dams were anesthetized with a mixture of xylazine (0.2 mg/g) and ketamine (0.5 mg/g) prior to receiving an intraperitoneal injection of oxytocin 20 UI/Kg (Vetnil® Ind. E Com. De Produtos Veterinários Ltda São Paulo, Brazil) to stimulate milk flow [50]. Fifteen minutes later, milk was obtained by hand stripping from all teats and collected into a Pasteur pipette, immediately stored in a microtube, and frozen at −80°C for later analyses. Total protein, glucose, and triglyceride concentrations were determined in all milk samples; samples were analyzed in duplicate and the values averaged. Milk protein was determined by the Bradford dye protein assay [51]. Milk glucose and triglyceride concentrations were determined using enzymatic colorimetric kits (Gold Analisa®, Belo Horizonte, Minas Gerais, Brazil). In addition, we also used cover glass methods to obtain leukocyte counts. Briefly, milk smears were air-dried and immediately stained with May-Grünwald (Bioclin/Quibasa® Belo Horizonte, Minas Gerais, Brazil). Differential leukocyte counts were performed by microscopic analysis of the stained preparations, using a Nikon Eclipse E200 Microscope (Nikon Corp., Tokyo, Japan) at a magnification of 100x. Ten microscopic fields were analyzed per section and one section per animal (six rats per group).

### 4.4. Milk Calories

For dosage of milk calories, a protocol established by Lucas et al. [52] was used. Briefly, 75 *μ*L of milk sample was collected in a capillary tube (triplicate) and immediately centrifuged for 15 minutes at 3000 rcf/minutes. After centrifugation, the cream and serum phases were measured in millimeters (mm) to estimate calorie content, according to an equation used by Brazil [53]. This equation is used for analyses of human milk caloric content by determining the following elements: (i)* cream content*: cream phase (mm) × 100/total phase (mm) = cream (%); (ii)* fat content*: cream (%) − 0.59/1.46 = fat (%); (iii)* total energetic content*: cream (%) × 66.8 + 290 = Kcal/liter.

### 4.5. Blood Biochemical Parameters and WAT Content

After milk collection, the dams were euthanized and their blood immediately was collected after decapitation. Serum was obtained by centrifugation and aliquots were stored at −80°C. Serum glucose and triglycerides were measured by colorimetric commercial kits (Gold Analisa, Belo Horizonte, Brazil) by means of an automatic analyzer (Selectra II, Bayer® Leverkusen, Germany). Serum insulin was analyzed by a radioimmunoassay technique (RIA). After euthanasia, the retroperitoneal and ovarian fat depots from dams were removed and weighed. In adult offspring, mesenteric adipose tissue depots were removed and weighed at 90 days of age.

### 4.6. Histology

After decapitation, MG (inguinal right), WAT (retroperitoneal and ovarian depots), tail of pancreas, and interscapular BAT tissues were prepared for histological analysis following standard techniques. Briefly, dissected tissues were fixed in 10% neutral buffered formalin (Merck, Buenos Aires, Argentina) during 72 h. Dehydration was performed by passing through ethanol solutions of increasing graduation (70, 80, 90, and 100%) and xylol and finally paraffin embedded. The tissues were sectioned at 5 *μ*m on a Reichert Jung rotary microtome (Leica RM 2025 Microsystems Inc., Wetzlar, Germany) and hematoxylin and eosin (H&E) stain was used for staining. Microscopic analysis of the stained preparations was carried out under an Olympus BX51 (Olympus microscope, Japan) and digital photographs were taken with a digital still 36-bit color camera (1280 × 1024 pixels), with a DP71 Controller (Olympus). Adipocyte numbers in MG were evaluated by individual adipocyte counts. In addition, adipocytes in the retroperitoneal and ovarian depots, as well as pancreatic islets, were used for the characterization of the cross-sectional area (size *μ*m^2^). The BAT proliferation was evaluated by nuclei counts. Approximately 4–8 microscopic fields per section and three sections per animal (six rats per group) were analyzed for all tissues using the Image J 1.42q software (Bethesda, MD, USA) available at the NIH site (http://rsb.info.nih.gov/ij, accessed on 12 April 2012).

### 4.7. Statistical Analysis

Data are expressed as means ± standard error mean (SEM). Statistical analyses were conducted using Prism for Macintosh, version 5.0 (Graphpad Software, San Diego, CA, USA). Statistical differences were evaluated by Student's *t*-test with *p* < 0.05 comparing SL Dams* versus* NL Dams and MSG Dams* versus* CON Dams. Qualitative histological analyses of the mammary gland were also performed.

## Figures and Tables

**Table 1:** Maternal metabolic parameters in blood from lactating dams at 21 days of lactation.

	NL Dams	SL Dams	CON Dams	MSG Dams
Glucose (mg/dL)	156.75 ± 5.80	209.20 ± 18.60^*∗*^	148.25 ± 2.40	200.00 ± 20.50^#^
Insulin (ng/mL)	0.55 ± 0.17	0.81 ± 0.26^*∗*^	0.33 ± 0.07	0.55 ± 0.10^#^
Triglycerides (mg/dL)	95.60 ± 7.00	137.43 ± 10.40^*∗*^	92.80 ± 11.20	160.60 ± 25.10^#^

Data are the means ± SEM obtained from 3–6 lactating dams per group. Symbols above numbers represent statistical differences (*p* < 0.05), according to Student's *t*-test. ^*∗*^NL Dams versus SL Dams; ^#^CON Dams versus MSG Dams.

**Table 2:** Milk composition in lactating dams at 21 days of lactation.

	NL Dams (*n* = 4)	SL Dams (*n* = 6)	CON Dams (*n* = 4)	MSG Dams (*n* = 5)
Calories (kcal/mL)	130.30 ± 10.67	172.30 ± 11.13^*∗*^	148.30 ± 9.64	143.70 ± 9.36
Fat content (%)	9.83 ± 1.10	14.28 ± 1.14^*∗*^	11.85 ± 0.99	11.34 ± 0.95
Protein (*µ*g/*µ*L)	69.80 ± 3.69	73.85 ± 6.29	73.34 ± 4.11	71.91 ± 2.61
Leukocytes/10 fields (1000x)	26.00 ± 2.17	22.80 ± 1.24	29.83 ± 3.61	45.83 ± 1.85^#^

Data are the means ± SEM obtained from 4–6 lactating dams per group. Symbols above numbers represent statistical differences (*p* < 0.05), according to Student's *t*-test. ^*∗*^NL Dams versus SL Dams; ^#^CON Dams versus MSG Dams.

**Table 3:** Body weight and visceral adipose tissue in adult SL and MSG offspring.

	NL offspring (*n* = 9)	SL offspring (*n* = 9)	CON offspring (*n* = 9)	MSG offspring (*n* = 10)
Body weight (g), 21 days	33.86 ± 1.38	39.29 ± 1.13^*∗*^	39.85 ± 0.99	39.13 ± 1.54
Body weight (g), 90 days	320.9 ± 7.55	344.7 ± 8.14^*∗*^	319.1 ± 9.62	282.2 ± 6.99^#^
Visceral adipose tissue (g/100 g)	0.68 ± 0.04	0.84 ± 0.04^*∗*^	0.71 ± 0.18	2.074 ± 0.05^#^

Data are the means ± SEM (*n* = 8–10 rats/group). Symbols above numbers represent statistical differences according to Student's *t*-test (*p* < 0.05). ^*∗*^NL offspring versus SL offspring rats; ^#^CON offspring versus MSG offspring rats.

## Abbreviations

SL: Small litter
NL: Normal litter
MSG: Monosodium glutamate
CON: Control
WAT: White adipose tissue
BAT: Brown adipose tissue
MG: Mammary gland
AUC: Area under curve
SEM: Standard error of the mean
mm: Millimeter
RIA: Insulin radioimmunoassay technique
H&E: Hematoxylin and eosin
UCP: Uncoupling protein.
